# Creating musical features using multi-faceted, multi-task encoders based on transformers

**DOI:** 10.1038/s41598-023-36714-z

**Published:** 2023-07-03

**Authors:** Timothy Greer, Xuan Shi, Benjamin Ma, Shrikanth Narayanan

**Affiliations:** 1 Signal Analysis and Interpretation Lab, University of Southern California, Los Angeles, CA 90089 USA; 2 Amazon Music, 525 Market St, San Francisco, CA 91405 USA; 3 Rivet App, 42 Whitman Street, MA 02144 Somerville, USA

**Keywords:** Psychology, Mathematics and computing

## Abstract

Computational machine intelligence approaches have enabled a variety of music-centric technologies in support of creating, sharing and interacting with music content. A strong performance on specific downstream application tasks, such as music genre detection and music emotion recognition, is paramount to ensuring broad capabilities for computational music understanding and Music Information Retrieval. Traditional approaches have relied on supervised learning to train models to support these music-related tasks. However, such approaches require copious annotated data and still may only provide insight into one view of music—namely, that related to the *specific* task at hand. We present a new model for generating audio-musical features that support music understanding, leveraging self-supervision and cross-domain learning. After pre-training using masked reconstruction of musical input features using self-attention bidirectional transformers, output representations are fine-tuned using several downstream music understanding tasks. Results show that the features generated by our multi-faceted, multi-task, music transformer model, which we call M3BERT, tend to outperform other audio and music embeddings on several diverse music-related tasks, indicating the potential of self-supervised and semi-supervised learning approaches toward a more generalized and robust computational approach to modeling music. Our work can offer a starting point for many music-related modeling tasks, with potential applications in learning deep representations and enabling robust technology applications.

## Introduction

The amount of consumable music has been growing rapidly over the past decades. As an effective way of utilizing such massive music content, automatically providing high-level descriptions about music (like genre, emotion, and theme) are becoming increasingly useful, which is why they are of interest to the MIR community^1,2^. Prior approaches have relied largely on supervised learning models^3–6^, which are trained on human-annotated music datasets. However, the performance of supervised learning is inherently limited by the size and scope of labeled music datasets, which can be prohibitively expensive and time-consuming to collect and generalize to new contexts and tasks. Recently, self-supervised pre-training models^7–10^, particularly Bidirectional Encoder Representations from Transformers (BERT), have been used extensively in the field of Natural Language Processing (NLP). BERT involves learning representations of language by reconstructing masked input sequences in pre-training. The intuition behind this design is that a model that can recover missing content of an input has learned a robust contextual representation of the input. BERT and its variants^11–13^ have achieved significant improvements on various NLP benchmark tasks^14^. Compared to the text domain, whose inputs are discrete word tokens, inputs are usually multi-dimensional feature vectors in the audio-acoustic domain: continuous and smoothly changing over time. Therefore, some particular designs have been introduced to bridge the gap between the original BERT model, which is trained on text, and audio-based transformer models, which are trained on acoustic data frames. Specifically for the domain of music audio, we use Contiguous Frame Masking (CFM) and Contiguous Channel Masking (CCM), as proposed in Zhao and Guo^15^, and compare it to Patch Masking, as done in Li et al.^16^. This model learns powerful acoustic music representations through pre-training. Finally, in order to adjust our model’s output representations for applications in downstream tasks, we fine-tune the outputs of this transformer model on several supervised music information retrieval relevant tasks at once. Because of the variety of the possible downstream tasks in the MIR community, creating a representation of music that is adaptable to diverse end tasks is important for model generalization and robustness. We use a multi-task learning approach to fine-tune the transformer-generated representations, ensuring that they are useful for broader music understanding. Our contributions are summarized below.

1. We present a new self-supervised pre-training model that is pretrained using diverse musical inputs and builds upon the structure of multi-layer bidirectional self-attention transformers; rather than relying on vast amounts of human-labeled data, this model can learn a powerful music representation from a variety of unlabeled music data. This model, which we will call M2BERT (multi-faceted, music BERT), is pretrained in a self-supervised fashion on audio data from 4281 h of music across four large and diverse music datasets.
2. We present several pre-training paradigms for M2BERT. Previous ablation studies have shown that a combination of CFM and CCM in tandem can effectively improve the performance of an audio-based transformer in pre-training^15^. In this work, we also use patch-masking and compare this paradigm to CFM and CCM.
3. We fine-tune our model on five diverse downstream tasks which span popular areas of research in MIR: genre classification, mood and theme detection, music emotion recognition (MER), and instrument classification. The final model, which we call M3BERT (multi-faceted, multi-task, music BERT), generates features that serve as better inputs for a variety of downstream music-related tasks, when compared to other commonly-used features. The success of M3BERT indicates the potential for applying transformer-based masked reconstruction pre-training (with subsequent multi-task enrichment) within the MIR field.
4. We conduct a correlational analysis with our encoder outputs, identifying certain cell activations that are similar to interpretable high-level audio features. This demonstrates that transformer models can generate features that are potentially human-understandable, lending to its appeal as a tool for music understanding and deriving meaningful music representations.

## Related work

### Transformer models

In the past few years, pre-trained models and self-supervised representation learning have yielded great success on NLP tasks. Many self-supervised pre-trained models based on multi-layer self-attention transformers^17^, such as BERT^18^, GPT^19^, XLNet^12^, and Electra^20^, have been used effectively. BERT is perhaps the most popular model due to its simplicity and outstanding performance across a variety of tasks. BERT reconstructs masked input sequences in its pre-training stage; through reconstruction, the model learns a powerful contextual representation of its input. More recently, the success of BERT in NLP has drawn attention from researchers in acoustic signal processing. Some pioneering works^7–10,21,22^ have shown the effectiveness of adapting BERT and other self-supervised approaches to Automatic Speech Recognition (ASR). By designing pre-training objectives specific to the audio modality, it is possible to adapt BERT-like models to music and other audio domains. In vq-wav2vec^21^, input speech audio is first discretized to a K-way quantized embedding space by learning discrete representation from audio samples. However, the quantization process requires heavy computing resources and runs counter to the continuous nature of acoustic frames. Other works^7–10,23^ have designed modified versions of BERT that directly utilize continuous speech. In some works^7,23^, and^8^, continuous frame-level masked reconstructions were adapted in a BERT-like pre-training stage. In other work^10^, SpecAugment^24^ was applied to mask input frames, and another method^7^ learned by reconstruction after shuffling acoustic frame orders rather than masking frames. Within the MIR realm, representation learning has been popular for many years. Several convolutional neural network- (CNN-) based supervised methods^3–6,25^ have been proposed for various music understanding tasks. These usually employ convolutional layers on Mel-spectrogram-based representations or raw waveform signals of music audio to learn effective music representations, and append fully connected layers to predict relevant annotations such as music genres or moods. However, training CNN-based models usually requires large datasets with reliable and consistent human-annotated labels. Other music representations have used contrastive learning^26–29^ for generating audio embeddings for downstream tasks. Carmon^30^ and Hendrycks^31^ have shown that using self-supervision on unlabeled data can significantly improve model robustness. More recently, self-attention transformers have shown promising results in music generation. For example, the Music Transformer^32^ and Pop Music Transformer^33^ employed relative attention to capture long-term structure from music MIDI data; however, compared with raw music audio, the size of existing MIDI datasets is limited. Transcription from raw audio to MIDI files is time-consuming and often not accurate, necessitating a transformer system that accepts (continuous) *audio input*. Other works have investigated lowering the computational cost of using transformers, potentially enabling greater model complexity and modeling capacity^28^.

### Multi-task learning

Multi-task learning (MTL) is an approach that involves assigning several tasks to a model to train on simultaneously^34^. This approach has been used to great extent in several music-related tasks, including frequency estimation^35^, source separation^36^ and instrument detection^37^. It is common for multi-task systems to favor well-represented tasks, sometimes at the expense of under-represented tasks^38^, and some research has attempted to ameliorate this problem^39,40^. As far as the authors know, self-supervised representations in music have not been fine-tuned on *multiple* music tasks, let alone tasks that span regression and classification. Ideally, musical features that show utility on several downstream music tasks simultaneously would be highly desirable for music research, providing a “one stop shop” to researchers attempting various tasks related to music understanding and MIR.

In this work, we propose M3BERT, a universal music-acoustic encoder based on transformers and multi-task learning. M3BERT is first pre-trained on large amounts of unlabeled music datasets, and then fine-tuned using an MTL approach on specific downstream music annotation tasks using labeled data.

## M3BERT model

**Figure 1:**
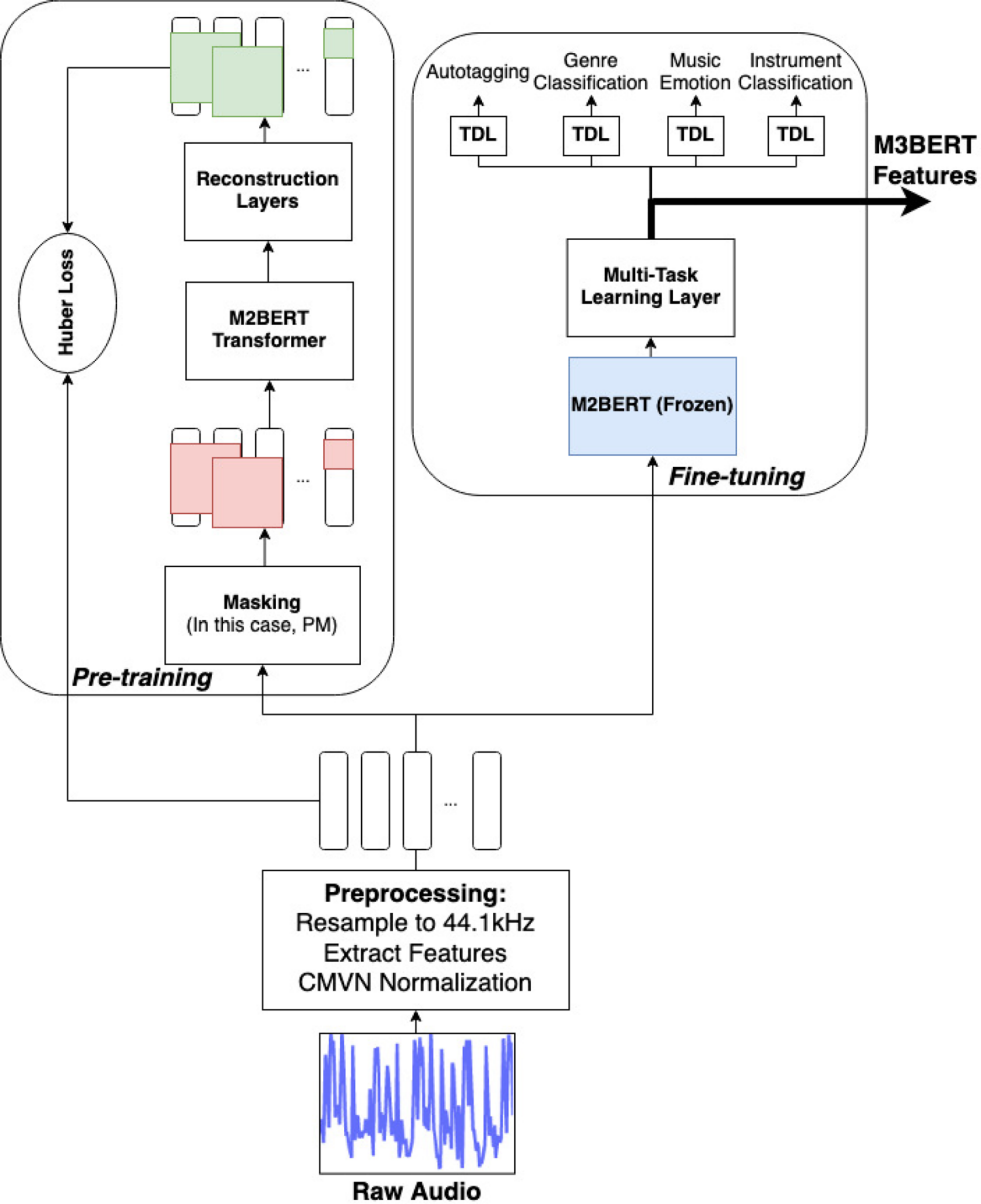
M3BERT pre-training and fine-tuning. During pre-training, the M3BERT transformer layers are updated and we use a Huber Loss between the reconstructed signal and the original signal. During fine-tuning, the M3BERT layers are frozen, and a dense, multi-task learning neural network layer is used to enrich the output representations. TDL stands for Time-Distributed layer, and without loss of generality, we show the patch-masking (PM) policy in this diagram.

**Figure 2:**
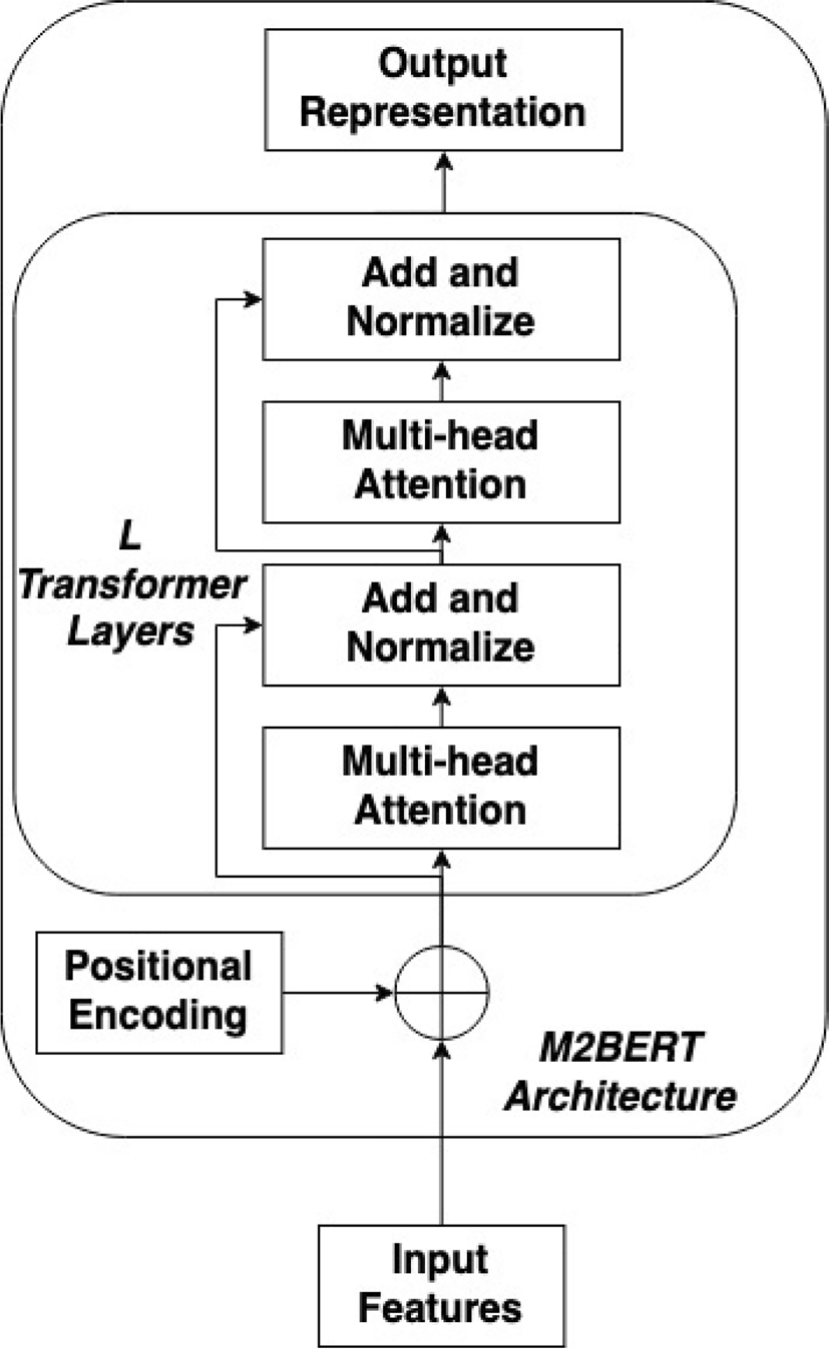
M2BERT architecture. M2BERT has *L* layers which use multi-head attention and normalization. This architecture, similar to BERT’s architecture, is used for pretraining; later a multi-task approach is used to enrich the output representations, providing a set of informative, interpretable features for downstream tasks.

A universal transformer-based encoder named M3BERT is presented for music representation learning. The system overview of the proposed M3BERT model is shown in Fig. 1, with details of the architecture listed in Fig. 2.

### Transformer encoder

A multi-layer bidirectional self-attention transformer encoder^17,18^ is used to encode input music frames, which are listed in Table 1. Specifically, an L-layer transformer is used to encode the input vectors $X = (x_i)_{i=1}^N$ as: $H^l = \text{ Transformer}_l(H^{l+1})$ where $l \in \{1,2 \ldots L\}$, $H^0 = X$, and $H^L = [h_1^L,...,h_N^L]$. We use the hidden vector $h_i^L$ as the contextualized representation of the input token $t_i$.

**Table 1:** Acoustic features of music extracted by Librosa^41^.

Feature	Characteristic	Dimension
Chromagram	Melody, Harmony	12
MFCCs	Timbre	20
Delta MFCCs	Timbre	20
Mel-scaled spectrogram	Raw waveform	128
Constant-Q transform	Raw waveform	144

We sought to use musical inputs that captured musical qualities such as timbre, melody, harmony, and spectrum (frequency-amplitude relationships).

### Pre-training and training

The main idea of masked reconstruction pre-training is to perturb inputs by randomly masking tokens with some probability and then using the model to reconstruct these masked tokens at the output. Intuitively, this is similar to dropout^42^, in which certain features or layers in a neural network are set to zero in order to prevent overfitting. In the pre-training process, a reconstruction module, which consists of two feed-forward layers with GeLU activation^43^ and layer-normalization^44^, is appended to the encoder-decoder architecture to predict the masked inputs. The multi-task system then uses the output of the last M3BERT encoder layer as its input. For clarity, we call M2BERT the *transformer* component of the overall model; M3BERT refers to the transformer with the additional multi-task layer of enrichment.

Several masking policies are presented for enabling M3BERT to learn music representations.

#### Masking policy 1: contiguous frame masking (CFM)

To prevent the model from exploiting local smoothness of acoustic frames, we mask spans of consecutive frames dynamically. Given a sequence of input frames $X = (x_1, x_2,\ldots , x_n)$, we select a subset $Y \subset X$ by iteratively sampling contiguous input frames (spans) until the masking budget (in this case, 15% of X) has been spent. At each iteration, a span length is first sampled from the geometric distribution $l \sim Geo(p)$. Then, the starting point of the masked span is randomly selected. We set $p = 0.2$, $l_{min} = 2$ and $l_{max} = 7$. The corresponding mean length of span is around 3.87 frames (179.6ms). Other schemes were also tried (variable lengths with different averages, constant lengths, etc.), but this scheme proved highest performance on downstream tasks. In each masked span, the frames are masked according to the following policy:

1. With 70% probability, replace all frames with zero. Since each dimension of input frames is normalized to have zero mean, setting the masked value to zero is equivalent to setting it equal to the mean.
2. Replace all frames with a random masking frame with 20% probability (mutually exclusive from 1).
3. Keep the original frames unchanged in the remaining cases (this happens 10% of the time). Since M3BERT will only receive acoustic frames without masking during inference time, this policy allows the model to receive real inputs during pre-training, resolving the pre-train/fine-tune inconsistency problem^18^.

#### Masking policy 2: contiguous channel masking (CCM)

The intuition of channel masking is that a model that can predict the partial loss of channel information has learned a high-level representation of such channels. For log-mel spectrum and log-CQT features, a block of consecutive channels is randomly masked to zero for all time steps across the input sequence of frames. Specifically, the number of masked channels, *c*, is first sampled from ${1,\ldots ,H}$ uniformly, where *H* is the number of total channels (in our case, this is 272). Then a starting channel index *h* is sampled uniformly from ${1,\ldots ,H-c}$ and the channels ${h,h+c}$ are masked.

#### Masking policy 3: patch masking (PM)

Often, music can be dynamic, quickly changing pitch, amplitude, and timbre. For this reason, it can be prohibitively difficult for a decoder to accurately reconstruct contiguous frames of features, particularly over long spans of music. Prior work in audio-based transformers has proposed patch masking^16^, which involves masking a square set of features (channels) and timesteps (frames). In the patch masking paradigm, squares of equal size are sampled with replacement until 15% of the input matrix is masked (see Fig. 1). We use this policy in comparison with a policy that uses CCM and CFM in tandem, which was found to be the best policy in a prior study^15^.

**Figure 3:**
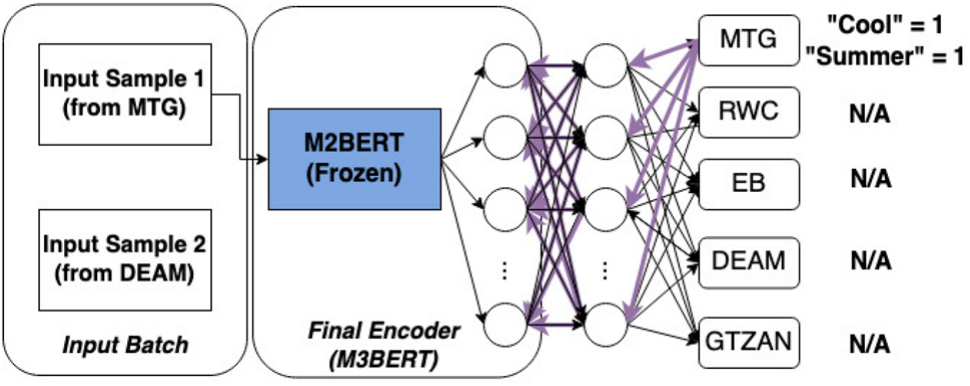
Multi-task learning on a sample from a batch. For this sample, there are only labels for the MTG-Jamendo task, so the weights for other tasks are frozen, as is M2BERT. We use cross-entropy loss for our classification tasks.

**Figure 4:**
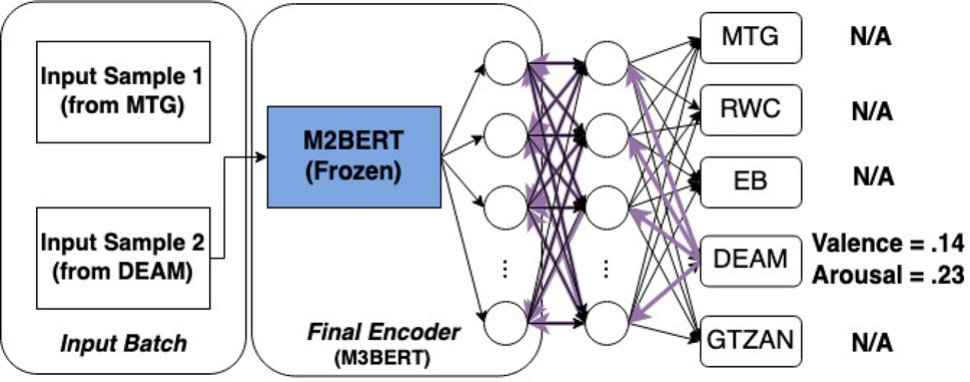
A workflow for another sample from the same batch. For this sample, there are only labels for the DEAM task, so the weights for other tasks are frozen, as is M3BERT. Weights are updated at the end of the batch. We use Mean Squared Error (MSE) loss for this regression task.

#### Pre-training objective function

1 $$\begin{aligned} \text{Huber}\;(\varvec{x},\varvec{y}) = {\left\{ \begin{array}{ll} 0.5|\varvec{x}-\varvec{y} |^2 &{} if~ |\varvec{x}-\varvec{y} |< 1\\ |\varvec{x}-\varvec{y} |- 0.5 &{} otherwise\\ \end{array}\right. } \end{aligned}$$

We use Huber loss^45^ to minimize the reconstruction error between masked input features and the corresponding encoder output. Huber loss is a robust $\ell _1$ loss that is less sensitive to outliers^46^. Additionally, a prior study^15^ found that using Huber loss made training converge faster than $\ell _1$ loss.

#### M3BERT model parameters

We report experimental results on two models: M3BERTSmall and M3BERTLarge. Model settings are listed in Table 2. The number of transformer block layers, the size of hidden vectors, and the number of self-attention heads are represented as $L_{num}$, $H_{dim}$, and $A_{num}$, respectively.

**Table 2:** Proposed model parameters.

	$L_{num}$	$H_{dim}$	$A_{num}$	Number of parameters
M3BERTSmall	4	768	12	29.3M
M3BERTLarge	8	1024	16	93.1M

## Methods

### Dataset curation and preprocessing

As shown in Table 3, the pre-training data were aggregated from four different datasets: Music4All^47^, FMA-Large^48^, MTG-Jamendo^49^, and Million Song Dataset^50^. Both the Music4all and FMA-Large datasets provide 30-s audio clips in mp3 format for each song. The MTG-Jamendo dataset contains 55,700 musical tracks, each with a duration of at least 30 s. Since the maximum sequence length of M3BERT is set to 1294 (30 s), music tracks exceeding this length are split up into 30 s chunks and treated as different samples. If a song is more than 30 s long but less than 60 s long, it is split up into two equal parts without overlap, as this ensures that every example is at least 15 s long and no more than 30 s long. This allows for more pre-training examples, along with potential bias: a long track may have more representation in the final embedding than a shorter song. As we have hundreds of thousands of training examples, accept the risk of skewed representation.

**Table 3:** Datasets used and statistics for pre-training and fine-tuning.

Task	Dataset	# Examples	Duration (h)
Self-supervised (pre-training)	Music4All	109.2K	908.7
Self-supervised (pre-training)	FMA-Large	106.3K	886.4
Self-supervised (pre-training)	MTG-Jamendo	55.7K	464.2
Self-supervised (pre-training)	Million Song Dataset	242.7K	2023.0
Genre classification (fine-tuning)	GTZAN	1K	8.3
Genre classification (fine-tuning)	Extended Ballroom	4.6K	38.3
Instrument recognition (fine-tuning)	RWC	12.9K	91.6
Music emotion recognition (fine-tuning)	DEAM	1.8K	18.3
Multi-label tagging (fine-tuning)	MTG-Jamendo	18.5K	157.1

The representations produced by the transformer are fine-tuned on five downstream tasks *in tandem* (see Figs. 3 and 4.): the GTZAN music genre classification task^51^, MTG-Jamendo music auto-tagging task^49^, Real World Computing (RWC) Instrument Classification task^52^, Database for Emotional Analysis of Music (DEAM) task^53^, and the Extended Ballroom task^54^ were all used to fine-tune M3BERT.

GTZAN consists of 1000 music clips divided into ten different genres (blues, classical, country, disco, hip-hop, jazz, metal, pop, reggae and rock). Each genre consists of 100 music clips in .wav format, each with a duration of 30s.

The MTG-Jamendo task consists of over 18,000 music clips, each with at least one mood or theme label. These genres range from common (“Happy” and the thirteen other most common tags are present in 68% of examples) to uncommon (the “Sexy” tag is present in .64% of samples) and the imbalance factor (the count of the most common tag divided by the count of the least common tag) is 15.7.

The Extended Ballroom dataset is an augmented version of the Ballroom dataset^55^. This dataset contains 4,180 music clips divided into 13 genres representing various ballroom dances (Cha Cha, Jive, Quickstep, etc). As these genres are closely related to rhythmic patterns, they can also be considered as rhythm classes. This dataset’s imbalance factor is also quite high, at 23 (Waltz is the most common label, and West Coast Swing is the least common). While other metadata is available (for example, artist and beats per minute of each song), we leave the possibility of leveraging such information for future work.

The RWC Musical Instrument Sound Database covers 50 musical instruments. At least three musicians played each instrument and at least three different manufacturers’ models were used for each instrument. To further provide a wide variety of musical instrument performances, the dataset includes samples from every tonal and dynamic range of each instrument.

After breaking long songs into smaller 30s chunks, the DEAM dataset consisted of 2099 excerpts annotated for overall (per-excerpt) emotional valence and arousal. Each sample was appraised for (perceived) valence and arousal by at least five annotators, and triplet embeddings of these labels were computed as in other studies^56,57^.

For GTZAN, we used the fault-filtered splits given in other literature^58^; for MTG-Jamendo, we organized the training, validation and testing sets as in previous literature as well^59^. For all other datasets, we could not find an agreed-upon set of splits in prior work, so we split up our data randomly into five equal parts, using three parts for training, one part for validation, and one part for testing. We split these data sets into equal parts according to number of songs in the original dataset. This policy ensures that excerpts from the same song are not present in training and testing after breaking up long songs into 30s chunks.

#### Audio preprocessing

The acoustic music analysis library Librosa^41^ was used to extract the following features from each song for pre-training: Mel-scaled Spectrogram, Constant-Q Transform (CQT), Mel-Frequency Cepstral Coefficients (MFCCs), Delta MFCCs and Chromagrams (see Table 1). Each feature was extracted at a sampling rate of 44,100 Hz, with a Hamming window size of 2048 samples (46 ms) and a hop size of 1024 samples (23 ms). The Mel Spectrogram and CQT features were transformed to log amplitude with $S_{new} = \ln (10~S +$ 1e-6), where *S* represents the original feature value. Then Cepstral Mean and Variance Normalization (CMVN)^60,61^ were applied to the extracted features to minimize the distortion caused by noise contamination. Finally, these normalized features were concatenated to form a set of 324 features per frame, which was later used as the pre-training input of M3BERT.

### Training setup

All of our experiments were conducted on 2 GTX 2080Ti. In pre-training, M3BERTSmall and M3BERTLarge were trained with an effective batch size of 128 for 200k and 500k steps, respectively. We applied an Adam optimizer^62^ with $\beta _1 = 0.9$, $\beta _2 = 0.999$ and $\epsilon = 10^{-6}$. The learning rate followed a warmup schedule^17^ according to the formula: $l_{rate} = \text{ min }(\frac{l_{max}s}{wT},\frac{l_{max}(T-s)}{T(1-w)})$ where *s* represents the step number, *w* represents the warmup steps (set to 7% of the total steps *T*), and $l_{max}$ represents the max learning rate (set to $2\cdot 10^{-4}$). For downstream tasks, we performed a grid search on a set of parameters and the model that performed best on the validation set was selected (see Table 4). All other training parameters remained the same as those in the pre-training stage.

**Table 4:** Parameter settings for downstream tasks.

Parameter	Candidate values
Batch size	16, 24, 32
Learning rate	2e−5, 3e−5, 4e−5
Epoch	2, 3, 4
Dropout rate	.05, .1

## Results

### Patch masking, CFM and CCM

We first survey the difference between patch masking, CFM, and CCM. When testing Patch Masking, CFM, and CCM individually on the MTG-Jamendo dataset, we find that Patch Masking outperforms the other two masking policies (Table 5.) However, when CFM and CCM are combined, as was conducted in a similar study^15^, the performance is better than Patch Masking. A hybrid approach of combining CCM, CFM, and Patch Masking simultaneously was not attempted because CCM and CFM already involves contiguous channel and frame masking. In subsequent results, we report on results that use CCM and CFM only. Experiments were conducted on the Jamendo dataset because it is the largest of the fine-tuning datasets and has canonical train-validation-test splits, allowing for seamless comparison to other approaches and masking policies^15.]^

**Table 5:** Performance of M2BERT on MTG-Jamendo using different masking policies.

Masking policy	ROC-AUC	PR-AUC
CCM	.6967	.0816
CFM	.7217	.0973
Patch Masking	.7308	.1073
CCM & CFM in tandem	**.7354**	**.1082**

Highest values for each metric are given in bold.

### Evaluation on downstream tasks

For each downstream task reported in the following sections, models using M2BERT and M3BERT embeddings were compared against models that use two commonly-used general-purpose audio features: MFCCs and VGGish embeddings. We also compared our representations against a contrastive learning approach on music, as implemented in previous work on Contrastive Learning of Musical Representations (CLMR)^26^. In addition, the state-of-the-art model performance using task-specific features and architectures is reported, if available.

### GTZAN

The test accuracy of the GTZAN dataset on the fault-filtered splits is shown in Table 6.

Although this small dataset is prone to overfitting^51^, the multi-task paradigm does not bring our results close to the performances of the state-of-the-art model, which pretrains a CNN on MSD and then finetunes the entire network on GTZAN, therefore qualifying as a deep end-to-end model.

**Table 6:** Results of a genre classification task on the GTZAN dataset.

Model	Accuracy (%)
MFCCs	44.8
VGGish^63^	53.8
M2BERT, no pretraining	56.1
M2BERT	60.1
M3BERTSmall	61.0
M3BERTLarge	61.7
Contrastive Learning of Musical Representations (CLMR)^26^	63.4
*CNN with pretraining*^64^	**82.1**

Approaches that use deep neural networks for prediction are italicized.

Highest value is given in bold.

### MTG-Jamendo emotions and themes in music

For the Jamendo mood-theme auto-tagging task, ROC-AUC macro and PR-AUC macro were used to measure performance. ROC-AUC can lead to over-optimistic scores when data is imbalanced^65^, and since the music tags given in the MTG-Jamendo dataset are highly imbalanced^66,67^, we also used PR-AUC for evaluation. The M3BERT model was compared with other state-of-the-art models from MediaEval 2020: Emotion and Theme Recognition in Music Using Jamendo^59^. We used the same train-validation-test data splits as the challenge. The results are shown in Table 7.

For the baseline model (based on VGGish features^63^) and the 2019 MediaEval winner^5^, we directly used the evaluation results posted in the competition leaderboard. For the 2020 winner^66^, we reproduced the work according to their implementation. This approach uses focal loss and CNNs to achieve state-of-the-art results. Our results suggest that improvement over past state-of-the-art work on this music auto-tagging task may be possible if a back-end architecture were to be used that integrates information over the temporal domain, such as a CNN. We applied a simple time-distributed dense layer to the output representations from M3BERT.

### Extended ballroom genre classification dataset

For the Extended Ballroom genre classification task, our performances were compared against other models, although the splits were different. Evinced by the best performing approach that does not use deep learning in Table 8, we see that rhythmic features appear to be helpful in predicting ballroom music genres, which were not used in our musical inputs. The best performing approach used a CNN-based model for genre prediction.

### DEAM music emotion recognition task

In the DEAM music emotion recognition task, our representations were compared against other feature sets, including VGGish features and MFCCs. In Table 9, we see that MFCCs perform poorly on this music emotion recognition task, while hand-crafted features and the more generalized VGGish features perform even better than our representations.

### RWC instrument detection task

In the RWC instrument classification task, our representations outperformed the other results found in the literature (see Table 10.) Understandably, timbral MFCC features perform better than VGGish features on instrument detection. It is evident here that representations are enriched in the multi-task stage, as performance is better using M3BERTLarge than using M2BERT.

**Table 7:** Results of an auto-tagging task on the MTG-Jamendo dataset.

Model	ROC-AUC	PR-AUC
MFCCs	.695	.081
VGGish^63^	.725	.107
M2BERT, no pre-training	.724	.104
M2BERT	.735	.109
CLMR^26^	.753	.108
M3BERTSmall	.777	.125
M3BERTLarge	.774	.125
*CNN (2019 Winner)*^5^	*.773*	*.155*
*CNN + Loss-function*^66^	**.781**	**.161**

Approaches that use deep neural networks for prediction are italicized.

Highest values per metric are given in bold.

**Table 8:** Results of a genre classification task on the Extended Ballroom dataset.

Model	Accuracy	Macro f1
MFCCs (our implementation)	.532	.381
MFCCs^25^$$^*$$	.623	–
M3BERTSmall	.704	.511
VGGish	.757	.602
M3BERTLarge	.812	.661
CLMR^26^	.830	.661
M2BERT, no pre-training	.817	.685
ConvNet Features^25^$$^*$$	.819	–
M2BERT	.820	**.685**
*Rhythmic Features + SVM*^68^$$^*$$	*.949*	–
*DenseNet*^69*^	**.967**	–

$^*$ indicates that the model evaluates on different subsets of the dataset than our work and hence numbers are not directly comparable. Approaches that use deep neural networks for prediction are italicized.

Highest values per metric are given in bold.

**Table 9:** Results of a music emotion recognition task on the DEAM dataset. $^*$ indicates that the model evaluates on different subsets of the dataset than our work and hence numbers are not directly comparable.

Model	$R_{V}^2$	$R_{A}^2$
MFCCs	.122	.327
CLMR^26^	.107	.384
M2BERT, no pre-training	.261	.515
M3BERTLarge	.266	.537
Hand-crafted Features^70^$$^*$$	.278	.529
M3BERTSmall	.332	.521
M2BERT	.345	.562
VGGish	**.395**	**.582**

Highest values per metric are given in bold.

**Table 10:** Results of an instrument detection task run on the RWC Instrument dataset.

Model	Accuracy	Macro-f1
Random Forest^71^$$^*$$	.549	–
Partials^72^$$^*$$	–	.634
CLMR^26^	.730	.717
VGGish	.821	.735
Cross-Dataset^73^$$^*$$	–	.823
MFCCs	.913	.875
M2BERT, no pre-training	.930	.898
M2BERT	.954	.933
M3BERTSmall	.951	.912
M3BERTLarge	**.966**	**.940**

$^*$ indicates that the model evaluates on different subsets of the dataset than our work and hence numbers are not directly comparable.

Highest values per metric are given in bold.

### Ablation study

Ablation studies were conducted to better understand the performance of M3BERT, similar to the work done by Zhao and Guo^15^. The results are shown in Table 11.

We removed datasets from pre-training to assess which datasets were most crucial to good performance on downstream tasks. Removing any dataset from pre-training results in a degradation in downstream performance on MTG-Jamendo autotagging; the larger the input dataset, the more severe the degradation. The multi-faceted music (M2BERT) model uses the diverse input datasets to inform its representations, and each dataset is evidently bringing a rich set of features for informing pre-training.

We also explore the effect that model size has on downstream task accuracy. In our experiments, M3BERTLarge generally outperforms M3BERTSmall, which remains consistent with the findings of Zhao and Guo^15^, although in tasks like valence prediction we see that M3BERTSmall outperforms M3BERTLarge. For other tasks, like mood-theme detection, we see comparable performance using either set of features. This suggests that for certain tasks, using the relatively economical M3BERTSmall features may be as effective as using M3BERTLarge features.

**Table 11:** Performance on MTG Autotagging with Ablation Study.

Missing Dataset	ROC-AUC	PR-AUC
MSD	.7058	.0874
FMA	.7216	.0977
M4A	.7234	.1006
MTG	.7267	.1035
None	**.7354**	**.1082**

Highest values per metric are given in bold.

### Correlational analysis

**Figure 5:**
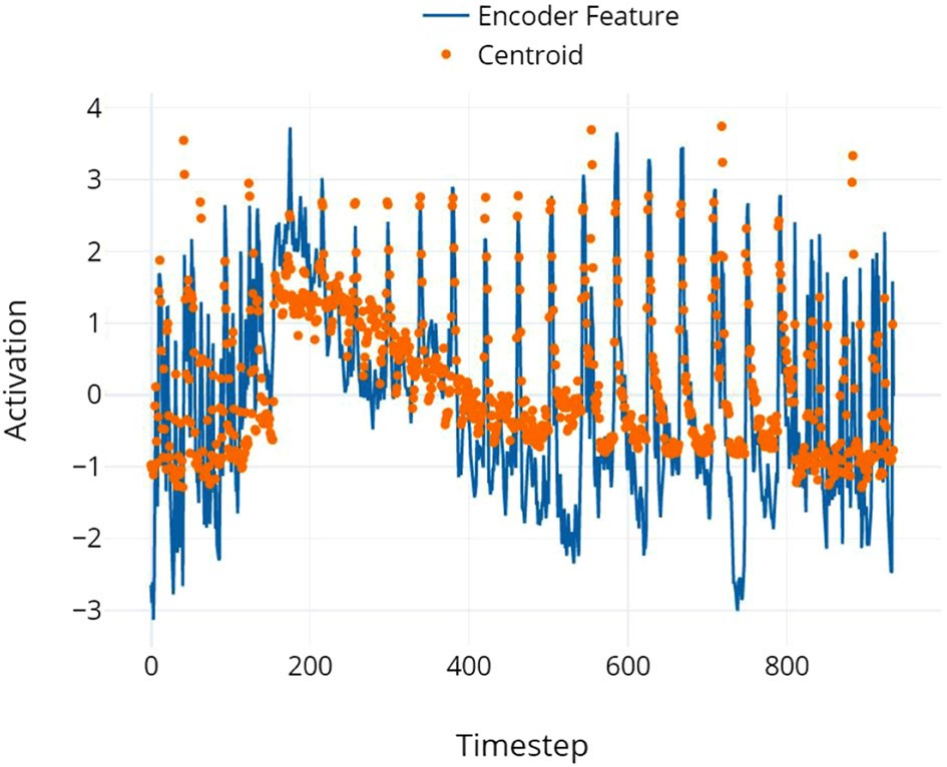
Centroid and cell activation. Certain outputs from the M3BERT encoder correlate highly with auditory phenomena, like spectral centroid. Pearson’s $\rho =.831$ between these two features.

**Figure 6:**
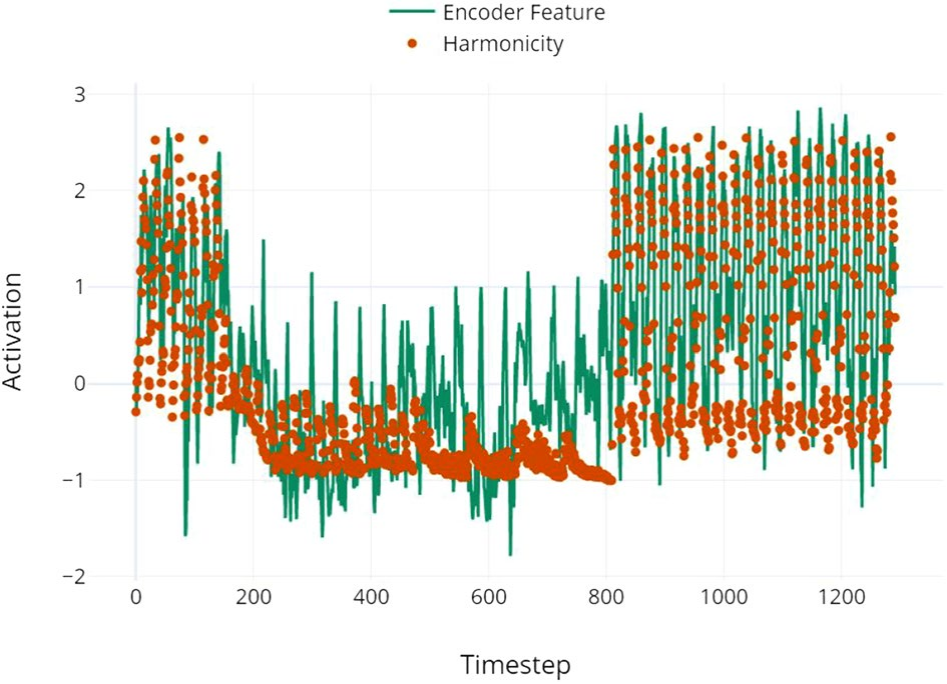
Harmonicity and cell activation. Interpretable auditory features like harmonicity were also correlated with certain outputs from M3BERT’s encoder. The encoder is creating high-level representations that are not necessarily based on frequency, as in this case. Pearson’s $\rho =.823$ between these two features.

Deep learning models and featuresets alike often suffer from a lack of interpretability^74^. In an effort to find representations of music that may be interpretable , we used Librosa^41^ to compute several high-level audio features, including brightness, loudness, and spectral flux. We then correlated these features with outputs from the M3BERT encoder. Results and correlations are shown in Figs. 5 and 6. We posit that these output representations from M3BERT are both powerful *and* interpretable , adding to their utility for studying music-related tasks.

## Discussion

We see that on several different types of downstream tasks, such as instrument detection and mood-theme autotagging, M3BERT produces features that, when passed through a simple neural network, post performance well better than other music features and—in the case of mood-theme autotagging—on par with the state-of-the-art model by the ROC-AUC metric. This makes M3BERT a useful first-stop-shop baseline for generating features for application to a diverse set of music-related tasks.

We observe that M3BERT performs much better on the mood-theme classification task than the M2BERT model: this may be because the multi-task learning paradigm exploited some labels that were present in the mood-theme detection task and the genre classification tasks. For example, one label in GTZAN is “jazz” and one label in MTG-Jamendo is “jazzy.” Curiously, the genre classification tasks did not benefit as much from multi-task learning; these datasets are relatively small compared to MTG-Jamendo, so in the multi-task paradigm, their samples are likely getting overwhelmed by the prevalence of MTG-Jamendo samples. We observe that performance on tasks with the least amount of training examples seems to degrade after multi-task training. While multi-task learning may not always improve the embeddings’ performance, with multi-task-specific loss function adjustments, such as those suggested by Kendall et al.^75^, it may be possible to improve on the results posted here.

In the classification and regression tasks, we averaged outputs across timesteps. This architecture was used for the sake of simplicity in creating representations of music, but it does not take advantage of the *temporal* dependencies of the musical inputs. If an architecture that captures this temporal information—such as a CNN or LSTM—were to be built upon the features that we created, we would expect to see greater improvement on these downstream tasks.

We see that although M3BERT performed very well on the instrument classification task, it did not perform as well on the GTZAN genre classification or DEAM music emotion recognition tasks. This may also be explained by the relative paucity of data (the MTG-Jamendo dataset is 18 times larger than GTZAN) and the input features we used for pre-training, which may not have spanned feature types that would be relevant for these prediction tasks. To wit, we used many features that related to *timbre*, which sensibly would perform well on an instrument classification task, but may not necessarily perform well on a music emotion recognition task, for example. Similarly, rhythmic features are shown to be effective in ballroom dance genre classification^68^, but were not represented in our initial input features. From our results, we hypothesize that choosing a broad set of input audio features and balancing fine-tuning across large, diverse datasets are important for creating robust representations of music.

We also note that a contrastive learning approach to creating music representations performs well on the genre detection tasks, outperforming M3BERT representations on the GTZAN dataset and the Extended Ballroom dataset. However, these representations seem to fall short on other tasks, especially the tasks related to music emotion recognition and instrument detection. We hypothesize that augmentations used during pre-training (on Magnatagatune^76^) do not translate well to music emotion recognition or instrument classification because positive pairs can have different arousal, valence, or sound quality, which could adversely affect embeddings used for related tasks.

In the interest of investigating interpretability of our embeddings, we present two high-level features that are highly correlated with outputs from M3BERT, including harmonicity and spectral centroid. While centroid is a rough measure for a song’s pitch, other frequency-based features were also correlated with cell activations, including brightness and spectral rolloff. Harmonicity and percussiveness were both correlated to encoder outputs ($\rho > .8$), and relate to timbre and, proximally, loudness (we did not analyze Root-Mean-Square of the waveform because it is captured in our encoder inputs by MFCC 0). Other features, including f0, spectral flatness and contrast, and zero crossing rate, were not found to be highly correlated with encoder outputs. These correlations suggest that certain base auditory features, like spectral centroid and harmonicity, are informative for a variety of music-related tasks; M3BERT may be used to uncover such features, providing MIR researchers additional insight into meaningful, interpretable features for tasks of interest.

## Conclusion

We propose M3BERT, a universal music encoder based on transformers. Rather than relying on massive human labeled data, which are expensive and time-consuming to collect, M3BERT can learn representations of music from unlabeled data and improve upon its representation with multi-task learning in fine-tuning. Contiguous Frame Masking, Contiguous Channel Masking, and Patch Masking are applied to the pretraining examples and features are created in reconstruction from a BERT-like, self-supervised transformer model. Subsequently, using a multi-task approach, this model enriches its features in a supervised manner, learning from several disparate music information retrieval tasks at once. The effectiveness of different masking policies, datasets, and input features are evaluated through ablation studies. We find that M3BERT outperforms commonly used features for music classification on a variety of music-related tasks, such as instrument classification and mood-theme detection . We also find that multi-task learning tends to enrich the representations generated by our encoder. Our work shows the potential of adapting a transformer-based, masked reconstruction pre-training scheme with multi-task learning to MIR interests. Beyond improving the model, we plan to extend M3BERT to other music understanding tasks, like key estimation and cover song detection, all while managing dataset imbalance to ensure that multi-task enrichment does not favor tasks with more examples. This work shows that marrying large-scale representation learning with diverse, supervised learning tasks can uncover powerful representations that can provide researchers a “canonical” first step to feature extraction for music-related tasks.

## Data Availability

Data used to train the M3BERT model can be found at http://millionsongdataset.com/, https://sites.google.com/view/contact4music4all, https://github.com/MTG/mtg-jamendo-dataset, and https://github.com/mdeff/fma. The datasets for fine-tuning M3BERT can be found at https://github.com/MTG/mtg-jamendo-dataset, http://anasynth.ircam.fr/home/media/ExtendedBallroom/, https://cvml.unige.ch/databases/DEAM/, https://www.tensorflow.org/datasets/catalog/gtzan, and https://staff.aist.go.jp/m.goto/RWC-MDB/rwc-mdb-i.html. Code for running scripts can be found at https://github.com/usc-sail/M3BERT.
